# Case-control Indian buffet process identifies biomarkers of response to Codrituzumab

**DOI:** 10.1186/s12885-019-5472-0

**Published:** 2019-03-28

**Authors:** Melanie F. Pradier, Bernhard Reis, Lori Jukofsky, Francesca Milletti, Toshihiko Ohtomo, Fernando Perez-Cruz, Oscar Puig

**Affiliations:** 1 Harvard University, Cambridge, MA USA; 2 Pharmaceutical Sciences, Roche Innovation Center Basel, Basel, Switzerland; 3 Translational Medicine Oncology, Roche Innovation Center New York, New York, USA; 4 pRED Informatics, Roche Innovation Center New York, New York, USA; 5 Chugai Pharmaceutical Co. Ltd., Tokyo, Japan; 6 Swiss Data Science Center, ETH Zurich, Switzerland; 7 Kite, a Gilead Company, Santa Monica, CA USA; 8 Translational Medicine, Bristol-Myers Squibb, Township, Lawrenceville, NJ USA; 9 University Carlos III in Madrid, Leganés, Spain

**Keywords:** Codrituzumab, Indian buffet process, Natural killer cells

## Abstract

**Background:**

Codrituzumab, a humanized monoclonal antibody against Glypican-3 (GPC3), which is expressed in hepatocellular carcinoma (HCC), was tested in a randomized phase II trial in advanced HCC patients who had failed prior systemic therapy. Biomarker analysis was performed to identify a responder population that benefits from treatment.

**Methods:**

A novel statistical method based on the Indian buffet process (IBP) was used to identify biomarkers predictive of response to treatment with Codrituzumab. The IBP is a novel method that allows flexibility in analysis design, and which is sensitive to slight, but meaningful between-group differences in biomarkers in very complex datasets

**Results:**

The IBP model identified several subpopulations of patients having defined biomarker values. Tumor necrosis and viable cell content in the tumor were identified as prognostic markers of disease progression, as were the well-known HCC prognostic markers of disease progression, alpha-fetoprotein and Glypican-3 expression. Predictive markers of treatment response included natural killer (NK) cell surface markers and parameters influencing NK cell activity, all related to the mechanism of action of this drug

**Conclusions:**

The Indian buffet process can be effectively used to detect statistically significant signals with high sensitivity in complex and noisy biological data

**Trial registration:**

NCT01507168, January 6, 2012

**Electronic supplementary material:**

The online version of this article (10.1186/s12885-019-5472-0) contains supplementary material, which is available to authorized users.

## Background

The cell surface heparan sulfate proteoglycan Glypican-3 (GPC3) is a serological and histochemical marker of hepatocellular carcinoma (HCC), due to its high and specific expression in HCC [1]. GPC3 promotes the growth of HCC by stimulating Wnt signaling [2], and GPC3 suppression inhibits growth of HCC cells via upregulation of TGF-β2 [3].

The anti-human GPC3 humanized monoclonal antibody Codrituzumab binds to GPC3 with high affinity [4] and interacts with CD16/FcγRIIIa, a receptor in Natural Killer (NK) cells to trigger antibody-dependent cytotoxicity (ADCC) [5]. Phase I studies in the US [6] and Japan [7] showed that Codrituzumab was well tolerated up to 20 mg/kg/wk. without dose limiting toxicity. These results led to a Phase II study to determine efficacy, in which Codrituzumab was well tolerated but did not meet pre-specified efficacy endpoints [8]. However, secondary analyses of data from the study found longer overall survival in patients with higher levels of Glypican-3 or CD16, indicating that a patient stratification strategy might improve outcomes.

In order to identify patients who might best respond to Codrituzumab, we conducted a retrospective analysis of biomarker data from the Phase II study, including demographic information, tumor histology, as well as serum and blood biomarkers. Variable drug exposure, observed in the treatment arm, confounded standard statistical approaches such as regression models [8]. Therefore, a novel probabilistic approach for clinical data analysis, the case-control Indian Buffet Process (C-IBP), was used. This method relies on the general latent feature model recently introduced in the machine learning literature [9].

The C-IBP identifies statistically significant biomarkers, both at a global and subpopulation level, by discovering a number of correlation patterns (referred to hereafter as latent features) among the observations, which might be present or absent for each patient individually. The method returns a list of representative subpopulations by grouping those patients that share the same set of latent features. Compared to other approaches, the number of correlation patterns and patient subpopulations does not need to be specified beforehand but is automatically learned from the data. This method also provides measures of uncertainty associated with each latent feature and subpopulation (e.g., a patient might stand between two subpopulations), as well as a method to isolate drug-related correlations from natural response patterns. The latent features provide a useful abstraction of the main properties of the data, which can be directly interpreted and analyzed by experts in the field, as shown in the subsequent analysis.

## Methods

### Study design and patients

Adult patients with unresectable advanced or metastatic HCC, previously treated with at least one line of systemic agent and with progressive disease, were enrolled in a randomized, placebo-controlled, double-blind, multicenter phase II trial (NCT01507168). Patients received either intravenous (IV) Codrituzumab at 1600 mg every two weeks (Q2W) or placebo (with a patient ratio of treatment:placebo of 2:1) until disease progression and were followed for overall survival. Details of study design have been previously described [8]. The study was approved by institutional review boards of participating centers and was conducted in accordance with the Declaration of Helsinki and Good Clinical Practice guidelines. NCT01507168 trial was approved by the institutional IRBs from all participating centers, and all patients received informed consent; it has already been published with a previous study in [8].

### GPC3 expression in tumor

All patients enrolled in the study provided a tumor tissue sample. Centrally reviewed immunohistochemistry (IHC) was used to determine the level of GPC3 expression in the tumor sample prior to study entry. IHC was performed on four 4 μm thick slides which were freshly cut from a formalin-fixed, paraffin-embedded block of the primary tumor (or the metastatic site) which had been obtained within approximately 12 months prior to informed consent. If no archival material was available, a pre-treatment core needle biopsy (using an 18-gauge or larger needle) was obtained and the sample fixed in formalin, embedded in paraffin and prepared as above. The IHC staining was done on BenchMark XT (Ventana Medical Systems, Inc. or VMSI, catalog number 750–700) or ULTRA (VSMI, catalog number 750–600) platforms. Each patient was assigned a GPC3 IHC score with ordered categorical values 0, 1+, 2+, and 3+, corresponding to increasing levels of GPC3 expression, with scores 0 and 3+ indicating the lowest and highest levels of GPC3 expression, respectively.

### Flow cytometry

Surface cell markers from circulating blood cells were measured by flow cytometry. Lymphocyte subsets were assayed using Trucount tubes (Becton, Dickinson and Company or BD, catalog number 340334). The expression level of CD16 on NK cells was measured by flow cytometry analysis of the pre-treatment peripheral blood mononuclear cells using a CD16-specific monoclonal antibody. Measurement was done on FACSCantoTM II (BD, catalog number 657338). CD16 expression level, or fluorescence intensity in units of Molecules of Equivalent Soluble Fluorochrome (MESF), denoted by CD16 MESF, was calculated by converting fluorescence measurements of the NK cell population to an MESF value based on an MESF calibration curve generated using the fluorescence intensity of calibration beads (QuantumTM MESF bead standard, manufactured by Bang Laboratories, IN, USA) [10].

### Soluble protein measurements

Monoclonal antibodies against soluble GPC3 protein were generated as previously described [11]. Five anti-N-terminal fragment mAbs (designated GT30, GT95, GT114, GT165 and GT607), and two anti-C-terminal fragment mAbs (designated GT57 and M3C11), were used in combination in four different assays (sGPC3 114/165, sGPC3 30/57, sGPC3 30/607, sGPC3 11/96) to detect full length GPC3 protein, or any possible cleavage fragments containing N- or C-terminus. The protocol for sandwich ELISA assay has been described [11].

### DNA polymorphisms

Genomic DNA was extracted from blood samples with a QiAmp Blood Mini Kit (Qiagen, Germany). DNA concentrations were measured with NanoDrop ND-1000 (Thermo Fisher Scientific, Wilmington, DE, USA), and DNA samples were diluted in nuclease free water to get a final concentration of 1 ng/μl. TaqMan technology on Applied Biosystems (AB) 7500 Fast Real-Time PCR system (Applied Biosystems Inc., CA, USA) was used to genotype patients for two different Fc gamma receptor polymorphisms, FcgRIIa-H131R and FcgRIIIa-V158F. We used probes and primers (TaqMan SNP Assays for rs1801274 and rs396991) from Applied Biosystems. Genotyping was performed following the manufacturer’s instructions.

### Statistical analysis

The Case-Control Indian Buffet Process (C-IBP) is an unsupervised approach to discover patient subgroups and common correlation patterns among the biomarker values. In particular, C-IBP finds a meaningful representation of patients by decomposing the data matrix X into the product of two matrices that are learned from data: a dictionary matrix B and a binary feature-activation matrix Z. Matrix B corresponds to the correlation patterns (also called latent features) that are most present in the data. Matrix Z indicates which features are active or absent for each patient individually.

In order to deal with the small sample-size scenario typical from clinical trials, C-IBP shares information between patients in the placebo and treatment arm. More particularly, C-IBP allows for two types of latent features: global features and treatment-specific features. Global features are learned from patients in the placebo arm, and can be active for any patient, capturing general patterns in the patient population, regardless of any treatment. In contrast, drug-specific features are learned from treated patients, and can only be active for patients in the treatment arm, capturing correlations linked to the effect of the drug.

The C-IBP approach was first used to identify subpopulations of patients having similar correlation patterns of biomarker values. The model describes each subpopulation using a specific signature of latent features that can be either present or absent. Progression Free Survival (PFS) for each subpopulation was monitored and groups with better survival outcome were identified. PFS was the clinical endpoint of the clinical trial. A biomarker analysis for each subgroup was conducted to identify statistically significantly associated biomarkers. Assessment of statistical significance for each biomarker was performed using the Mann Whitney test for continuous variables and the Fisher test for discrete variables. In order to account for multiple hypothesis testing we followed the Benjamini and Hochberg procedure to estimate False Discovery Rate and adjust statistical significance levels [12]. An illustration of the whole pipeline and further details can be found in the Additional file 1.

## Results

180 patients (120 in the treatment arm, 60 in the placebo arm) with HCC were enrolled in a randomized, placebo-controlled, double-blind, multicenter phase II trial of Codrituzumab. The primary efficacy endpoint was not met, and when a population pharmacokinetic (PK) analysis was performed, a highly varied range of drug exposure in the treatment arm was observed, resulting in only half of the patients receiving appropriate drug exposure [8]. In order to identify subgroups of patients benefiting from the drug, we performed a comprehensive retrospective biomarker analysis using standard techniques (list of biomarkers shown in Additional file 2 and biomarker data in Additional file 3), however, standard statistical approaches did not find any significant biomarkers, probably because of the variable and heterogeneous nature of the data [8]. Alternatively, the C-IBP is a novel probabilistic approach to clinical data analysis which was able to deliver statistically significant biomarkers.

### Latent feature analysis

We used Progression Free Survival (PFS) as a clinical endpoint in our analysis. As shown in Table 1, the C-IBP model identified twelve subpopulations from the set of 180 patients, and three latent features (F1, F2, and F3) which capture correlation patterns of biomarker values. These features can be either present (=1) or absent (=0) for each patient individually. A feature is present when the corresponding pattern contributes to the total biomarker values of that patient. A subpopulation is defined as a group of patients having similar biomarker values, encoded by the same set of present features. The C-IBP model is constrained to identify two types of features: global features, which can be active for any patient (F1 and F2), and drug-specific features, which can only be active for patients in the treatment arm (F3). The number of each type of latent feature was not fixed beforehand but learned from data.

**Table 1:** Subpopulations discovered by the Case-Control Indian Buffet Process (C-IBP)

Sub-population	Drug Identifier	F1	F2	F3	Average(*) num. of patients	Mean PFS (month)	Median PFS (month)
1.	0	0	0	0	33.37	3.06	1.65
2.	0	0	1	0	4.07	2.29	2.24
3.	0	1	0	0	17.84	2.72	1.81
4.	0	1	1	0	4.72	7.05	7.18
5.	1	0	0	0	51.52	3.22	2.55
6.	1	0	0	1	16.77	4.17	3.65
7.	1	0	1	0	8.38	1.74	1.33
8.	1	0	1	1	2.07	2.69	2.65
9.	1	1	0	0	29.88	3.36	2.03
10.	1	1	0	1	4.90	4.44	4.34
11.	1	1	1	0	4.53	6.31	5.31
12.	1	1	1	1	1.94	10.04	10.01
Total	120.0	63.82	25.72	25.69	180	3.44	2.04

Three latent features (F1, F2, F3) describe the population in this study, and each subpopulation can have the feature active (1) or inactive (0). Mean PFS was used as meaningful clinical endpoint. (*) Average number of patients is reported since the C-IBP is a probabilistic method that allows for soft-clustering of the patients (each patient belong to a subpopulation with a certain probability)

Tables 2, 3, 4 contain the biomarkers that are statistically significant in relation to each of the three features (F1-F3). The sign (+ or -) indicates the direction of the biomarker effect in the case group versus the control group, such that positive effect means that the biomarker has a higher value in the case group versus the control group. Feature F1 is represented by high levels of alpha-fetoprotein and GPC3 expression both in tumor (GPC3 cytoplasmic and membrane IHC staining) and as soluble protein sGPC3 (as detected by four serum assays), as shown in Tables 2, 3, 4 and Additional file 4). Feature F2 is associated with higher levels of inflammatory T and NK cells (positively stained for CD3/CD16) in tumor necrotic tissue and adjacent peri-tumoral stroma, and low levels of CD3/CD16 in viable tumor cells (Table 3 and Additional file 4). The combination of F1 and F2 is associated with better prognosis. Feature F3, the feature associated with drug treatment, is associated with higher levels of T (CD3, CD45) and NK (CD16, NKp46) cell markers (Table 4 and Additional file 4).

**Table 2:** Biomarkers significant for feature F1, ranked by effect size

Continuous Variables	Effect Size	*p*-value
AFP	3.65	1.31e-07
GPC3 membrane	1.30	1.53e-21
GPC3 cytoplasmic	0.69	2.08e-16
sGPC3 11/96	0.47	1.34e-04
sGPC3 30/57	0.40	1.53e-05
sGPC3 30/607	0.34	6.21e-10
sGPC3 114/165	0.32	2.48e-10

AFP, alpha-fetoprotein; GPC3 membrane, GPC3 expression, as measured by H score in cell membrane; GPC3 cytoplasmic, same as before, but measure in cell cytoplasm; sGPC3_NN, are the different soluble assays against serum GPC3

**Table 3:** Biomarkers significant for feature F2, ranked by effect size

Continuous Variables	Effect Size	*p*-value
CD3/CD16 necrotic	3.94	1.29e-17
%Necrotic	3.91	9.39e-18
CD3/CD16 viable	−1.27	1.29e-17
%Viable	−1.24	9.39e-18

CD3/CD16 necrotic/viable, staining for CD3 and CD16 in the necrotic tissue and viable cells respectively; %Necrotic/%Viable: percentage of necrotic or viable cells in tissue; AAT: Alanine Aminotransferase. Note that CD3/CD16 necrotic is the count of CD3 CD16 double positive cells in the necrotic tissue and stroma tissue counted together

**Table 4:** Biomarkers significant for feature F3, ranked by effect size

Continuous Variables	Effect Size	*p*-value
CD56dimCD16bright	1.27	2.18e-09
NK	1.24	2.13e-10
CD56-CD16+	0.61	2.19e-06
CD56dimCD16-	0.42	1.04e-05
CD8 NK	0.37	7.13e-08
DN	0.27	3.73e-05
CD16	0.24	6.06e-10
NKP46	0.22	1.14e-09
CD8	0.20	3.44e-09
CD45	0.14	5.09e-11
CD3	0.13	1.49e-08
CD4	0.12	5.06e-06

Feature F3 is represented by NK and T cell subpopulations. Hazard Ratio was 0.75 with 95% confidence interval [0.57; 1.00]. See Additional File 4 for details

### Analysis of subpopulations

One of the outstanding properties of the C-IBP method is its flexibility in analyzing complex datasets, allowing a detailed analysis of subpopulations by using shared features to group them. For example, in the placebo arm, the C-IBP model found (learned from the data) four subpopulations (groups 1 to 4 in Table 1). Subpopulation 4 has a much higher PFS compared with the other three subgroups, indicating better prognosis in the absence of treatment. Out of the 70 variables analyzed simultaneously by the C-IBP method, higher levels of inflammatory T and NK cells (CD3/CD16) in tumor necrotic tissue and adjacent peri-tumoral stroma, and lower levels in viable cells in tumor (CD3/CD16 staining in viable tumor cells) correlate with better prognosis (Table 5).

**Table 5:** Biomarkers significant in subpopulation 4, ranked by effect size

Continuous Variables	Effect Size	*p*-value
%Necrotic	2.73	1.86e-04
CD3/CD16 necrotic	2.63	2.85e-04
%Viable	−0.86	1.86e-04
CD3/CD16 viable	−0.85	2.85e-04

Biomarkers significant for subpopulation 4 (subjects with longer PFS in placebo group), compared to subpopulations 1, 2 and 3 (subjects with short PFS in placebo group), ranked by effect size. Biomarkers as in Table 3

In the treatment arm (groups 5 to 12 in Table 1), subpopulations with feature F3 active (6, 8, 10 and 12) have higher PFS values compared with the similar subpopulation in which F3 is not active. The effect of F3 on prolonged survival was found across all subpopulations, as seen by pairwise comparisons between subpopulations in the treatment arm with F3 active vs. the ones with F3 inactive (eg. 5 vs 6, 7 vs 8, etc). Table 6 shows that biomarkers correlating with F3 in the treatment group are similar to the ones correlating with F3 globally (Table 4), i.e., higher levels in blood of inflammatory T (CD3, CD45) and NK (CD16, NKp46) cells.

**Table 6:** Biomarkers significant for feature F3 in the treatment arm, ranked by effect size

Continuous Variables	Effect Size	*p*-value
CD56dimCD16bright	1.36	1.76e-09
NK	1.33	9.51e-11
CD56-CD16+	0.66	1.26e-06
DP	0.59	2.14e-04
CD56dimCD16-	0.44	4.39e-06
CD8 NK	0.39	9.50e-08
DN	0.30	3.58e-05
CD16	0.25	3.36e-10
NKP46	0.25	3.29e-10
B	0.21	2.29e-04
CD8	0.21	2.67e-09
CD45	0.15	3.75e-11
CD3	0.14	1.00e-08
CD4	0.14	1.48e-06

Feature F3 in the treatment group is represented by NK and T cell subpopulations. See Additional file 4 for details

### High drug exposure analysis

Patients received intravenous Codrituzumab at a dose of 1600 mg every two weeks (Q2W), however, drug exposure measurements showed that drug levels varied considerably among patients. We therefore repeated the biomarker statistical analysis using biomarker data only from patients with high Codrituzumab exposure (above median) compared to placebo. Table 7 (to be compared with Table 1) shows the 12 subpopulations and 3 features found by the C-IBP approach when excluding patients with low drug exposure. Features F1, F2 and F3 (Additional files 5, 6, 7) were characterized by sets of biomarkers similar to those identified in the all-patient analysis, indicating that each major group characteristics are independent of drug exposure, and are intrinsic to the subjects in each group.

**Table 7:** Subpopulations discovered by the C-IBP, only considering the high exposure group and placebo group

Sub-population	Drug Identifier	F1	F2	F3	Average num. of patients	Mean PFS (months)	Median PFS (months)
1.	0	0	0	0	33.16	3.07	1.65
2.	0	0	1	0	4.17	2.30	2.21
3.	0	1	0	0	17.96	2.70	1.81
4.	0	1	1	0	4.71	7.02	7.15
5.	1	0	0	0	25.67	4.15	3.93
6.	1	0	0	1	6.72	4.64	4.23
7.	1	0	1	0	4.23	2.96	2.29
8.	1	0	1	1	1.37	3.69	3.67
9.	1	1	0	0	14.63	3.62	2.66
10.	1	1	0	1	1.55	5.94	5.93
11.	1	1	1	0	3.86	6.23	5.37
12.	1	1	1	1	1.98	10.63	10.62
Total	60.00	44.69	20.31	11.61	120	3.78	2.76

The values are similar to those in Table 1, corresponding to the analysis performed in the entire cohort

C-IBP deliberately includes all patients in order to learn a joint meaningful patient representation via latent features that manifest differently for different patients. The inclusion of patients that received low drug exposure allows discovering prognostic factors (in latent feature F1 and F2), as well as drug-specific effects (in latent feature F3) by sharing information (e.g., occurrence of correlation signatures) across patients with varying levels of drug exposure. Table 7 and Additional files 5, 6, 7 confirm the statistical significance of the discovered biomarkers by performing two-sample tests only on patients who received high drug exposure (but after having found a latent projection based on information from all patients).

## Discussion

The identification of biomarkers in complex datasets affected by multiple confounding factors can be challenging. In drug development, clinical trials are powered to deal with situations of constant drug exposure, and traditional statistical methods have difficulties extracting signals from data confounded by factors like differential drug exposure. Therefore, sensitive analytical methods are needed to deconvolute real signals from biological and technical variability. We applied the Case-control Indian Buffet Process (C-IBP) to a phase II clinical study of Codrituzumab in HCC which had failed to meet the primary endpoint due to insufficient drug exposure in the treatment arm [8]. Beyond GPC3 and CD16 expression, traditional statistical approaches did not render useful insights into potential biomarkers of response [8]. In contrast, the C-IBP analysis identified several biomarkers that stratified patient subgroups with statistical significance, and meaningful biology.

While variable drug exposure confounded previous statistical approaches such as regression models [8], C-IBP was able to unravel statistically significant biomarkers despite of the drug exposure confounder, which makes it particularly attractive and suitable for this kind of data. C-IBP learned a meaningful latent representation of patients by sharing information from all patients. The learned features were used to identify homogeneous subgroups of patients for whom classical statistical analyses were performed. Nonetheless, we remark that, although all statistical tests were independent, we cannot rule out all confounding effects across subgroups.

The C-IBP model identified two kinds of features: global features, which were active for patients regardless of treatment, and indicated prognostic markers, and drug-specific features, which were only allowed to be active for patients in the treatment arm, and were therefore linked to drug response. Global prognostic features included known prognostic markers in HCC, like alpha-fetoprotein [13] and GPC3 expression in the tumor [1]. In addition, global features included levels of inflammatory T and NK cells in tumor necrotic tissue and adjacent peri-tumoral stroma. In this regard, patients with higher levels of T and NK cells (identified by CD3/CD16 staining) in peri-tumoral stroma and lower levels in viable tumor cells had better prognosis, which is consistent with the role of these inflammatory cells in anti-tumor response [14, 15]. Drug-specific features included different NK cell subtypes, which confirms previous findings of the central role for NK cells in HCC [16]. Specifically, a reduction of blood CD56dimCD16bright NK cells has been correlated with poor prognosis in HCC [17], and we observed that presence of this subset was the most predictive of positive drug response, both in the entire study and in the high exposure group (Tables 4 and 6). These findings are consistent with the mode of action of Codrituzumab, which requires engagement of the CD16/FcγRIIIa receptor in NK cells to recruit NK cells to the tumor with subsequent tumor lysis [5].

## Conclusions

In summary, the C-IBP approach applied to a complex Phase II clinical study in HCC confounded by several factors was able to identify prognostic and predictive biomarkers of response to Codrituzumab. The C-IBP method is flexible, as it automatically infers the required number of latent features that best explain the observations. We were able to identify both prognostic and predictive variables, as well as quantify the direction of action, effect size and statistical significance for each biomarker. Our model handles data variability and missing information naturally. In order to deal with the small sample-size problem, C-IBP shares information among patients (by defining global features active for any patient, or drug-specific features, that are constrained by model design to eventually activate only for patients having taken the drug). By following such approach, there is a clear separation between drug effects and natural prognostic factors.

## Additional files

Additional file 1: Method description. (PDF 335 kb)

Additional file 2: List of biomarkers in this study and abbreviations. (XLSX 7 kb)

Additional file 3: Biomarker and clinical endpoint data. (CSV 51 kb)

Additional file 4: Relative effect size of biomarkers associated to each latent feature inferred by the C-IBP model. Significant biomarkers according to the Mann-Whitney test are marked with red circles. F1 identifies two types of patients with similar prognosis but different characteristics, F2 and F3 are associated with higher Progression Free Survival: F2 capture prognostic biomarkers while F3 capture predictive biomarkers. (PDF 85 kb)

Additional file 5: Biomarkers significant for feature F1 when only considering patients with high exposure and placebo. (XLSX 6 kb)

Additional file 6: Biomarkers significant for feature F2 when only considering patients with high exposure and placebo. (XLSX 6 kb)

Additional file 7: Biomarkers significant for feature F3 when only considering patients with high exposure and placebo. (XLSX 6 kb)

## Abbreviations

ADCC: antibody-dependent cytotoxicity (ADCC)
C-IBP: Case-control Indian Buffet Process
GPC3: Glypican-3
HCC: Hepatocellular carcinoma
IBP: Indian Buffet Process
IHC: Immunohistochemistry
MESF: Molecules of Equivalent Soluble Fluorochrome
NK: Natural killer
PFS: Progression Free Survival
