# Supplementary material for: Case-control Indian buffet process identifies biomarkers of response to Codrituzumab
Source: BMC Cancer. 2019 Mar 28;19:278. doi: 10.1186/s12885-019-5472-0 (PMC6438135; doi:10.1186/s12885-019-5472-0)
Supplement: Supplementary file 1 — Method description. (PDF 335 kb) [file 12885_2019_5472_MOESM1_ESM.pdf]

Additional File 1: Method Description

1 Problem Statement

Let N be the total number of patients involved in the clinical trial, and D the number of observed variables per patient, including all biomarker values. Let $\mathbf{X}$ be the $N \times D$ data matrix gathering all available information for each patient row-wise. Such matrix might be incomplete, noisy and very heterogeneous, i.e., columns of $\mathbf{X}$ (screened variables) might correspond to different types of data, including continuous, positive real-valued, categorical, ordinal or count data. Among the N patients, we count N_p patients in the placebo arm and N_t patients in the treatment arm. Let $\mathbf{R} = \{r_n\}_{n=1\ldots N}$ be a binary drug identifier vector which takes non-zero values for patients belonging to the treatment arm, e.g., $r_n = 1$, and zero values for patients belonging to the placebo arm. Among the D dimensions, we have a variable d^* that captures how well patients are doing, e.g., time of Progression Free Survival (PFS). In such scenario, our objective is to discover prognostic and predictive biomarkers with respect to d^*, i.e., prognostic variables help us predict the natural evolution of patients regardless of the treatment, while predictive variables allow us to anticipate patient drug responses.

2 Probabilistic Model

General Latent Feature Model In order to analyze the data, we resort to a latent feature model, an unsupervised approach that models the probability of all available data $p(\mathbf{X})$ jointly, using a set of latent features [6]. Each latent feature captures common correlation patterns among the dimensions, and the objective is to learn the most probable set of such latent features[1]. Figure 1 illustrates the idea underlying a latent feature model. $\mathbf{X}$ can be decomposed into the product of two matrices that should be learned from data: a feature-activation matrix $\mathbf{Z}$ and a dictionary matrix $\mathbf{B}$. Each element $x_{nd} \in \mathbf{X}$ results from a linear combination of K feature elements B_{kd}, i.e., x_{nd} corresponds to the realization of a random variable following the probability distribution $f(\mathbf{Z}_n\mathbf{B}_d)$, where $\mathbf{Z}_n$ is the n-th row of $\mathbf{Z}$ and $\mathbf{B}_d$ is the d-th column of $\mathbf{B}$.

In our particular case, we use the General Latent Feature Model (GLFM) first introduced in [8] and further described in [7, 9], which improves upon classical latent factor models in three aspects. First, it is a Bayesian non-parametric

[1] More precisely, the objective is to learn a posterior distribution for each latent feature.

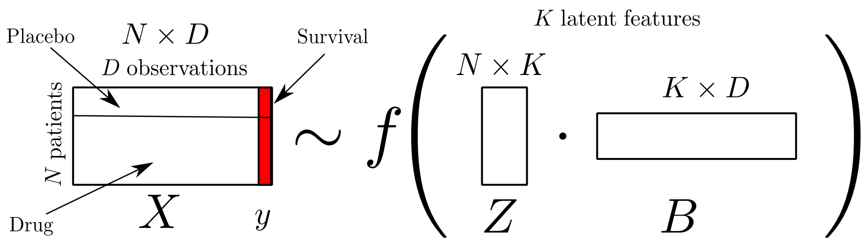

Figure 1: **Illustration of the matrix factorization scheme.** $\mathbf{Z}$ is the feature-activation matrix, $\mathbf{B}$ is the dictionary matrix, and f is the model likelihood which depends on the type of data.

model where the number of latent features is also learned from data [2, 3]. In other words, the model assumes an a priori unbounded number K of latent features, usually denoted by $K \to \infty$, which is an useful property, given that the number of correlation patterns to be discovered is generally not known beforehand.

Second, the GLFM can handle heterogeneous datasets and missing observations straightforwardly[2]. This comes handy to deal with clinical trial data, where observations for each patient are typically diverse in nature, and missing values occur frequently (e.g., not all patients might get the same tests run, others might drop out from the study at some point, etc).

The third advantage of the GLFM is that it allows for a partition of the patients in different sub-populations. The model assumes a binary feature-activation matrix $\mathbf{Z} \in \{0,1\}^{N \times K}$, which admits an easy interpretation in which each latent feature can be either active or inactive for each patient. Patient sub-populations can then be identified by gathering all patients that have the same set of active features. Within the Bayesian framework, the GLFM assumes a Gaussian prior for each element of $\mathbf{B}$, and it resorts to the Indian Buffet Process (IBP) as a prior for the feature-activation matrix $\mathbf{Z}$ [8].

Indian Buffet Process The IBP is a stochastic process which defines a probability distribution over binary matrices with a finite number of rows and unbounded number of columns [3]. It is often illustrated using a culinary metaphor that gives the name to the process. Imagine an Indian restaurant whose buffet consists of infinitely many dishes arranged in a line. N customers enter the restaurant sequentially. The first customer starts at the left of the buffet and takes a serving from each dish, stopping after a Poisson(α) number of dishes, as his plate becomes overburdened. The n-th customer moves along the buffet and samples dishes in proportion to their popularity, serving himself with probability $\frac{m_k}{n}$, where m_k is the number of previous customers who have sampled dish k. Having reached the end of all previously sampled dishes, the n-th customer then tries a Poisson($\frac{\alpha}{n}$) number of new dishes. In our case, each customer

[2]In the GLFM, the likelihood f will vary column-wise depending on the type of data.

corresponds to a patient, and each dish corresponds to a certain latent feature or condition that can be active or inactive for each patient.

Case-control Indian Buffet Process In order to deal with the small sample-size scenario typical from clinical trials, we adapt the IBP in order to share information between patients in the placebo and treatment arm. In particular, we allow for two types of latent features: *global* features and *treatment-specific* features. Global features are learned from patients in the placebo arm, and can be active for any patient, capturing general patterns in the patient population regardless of nay treatment. In contrast, drug-specific features are learned from treated patients, and can only be active for patients in the treatment arm, capturing correlations linked to the effect of the drug. We call this extension the Case-control Indian Buffet Process (C-IBP).

The learning algorithm to train the C-IBP model can be easily described as a two-step procedure directly based on the inference of GLFMs [8]. We first learn the global features by training the GLFM with patients belonging to the placebo arm exclusively[3]. Next, we learn the drug-specific features based on patients in the treatment arm. This is performed by training the GLFM model with the whole patient population and imposing the constraints listed in Alg. 1. Note that the treatment-specific features are always inactive for patients in the placebo arm, which allows to completely isolate the effect of the drug. Alg. 1 describes this procedure. Code to train the C-IBP model can be found publicly available online at the authors webpage.

Algorithm 1 Inference procedure for the C-IBP.

1: Train a GLFM using patients from the placebo arm only. We thus learn a set of global features, as well as the feature assignments for patients in the placebo arm. Inference is fully described in [8].

2: Train a GLFM using all patients. Inference also follows from [8], combined with the additional three constraints:

- (i) global features are kept fixed to the values learned in previous stage.
- (ii) feature assignments for patients in the placebo arm are initialized to their value in previous stage.
- (iii) treatment-specific features are forced to be inactive for all patients in the placebo arm, i.e., treatment-specific features are learned solely based on patients belonging to the treatment arm.

[3]Code for the GLFM can be found publicly available in matlab, R, and python at: https://github.com/ivaleraM/GLFM.

3 Statistical Methodology

Once the model has been trained (samples from an approximate posterior distribution can be drawn), we proceed with a classical frequentist approach[4] to identify statistically significant prognostic and predictive biomarkers. The whole procedure is summarized in Algorithm 2. First, we take M posterior samples from the posterior distribution of $\mathbf{Z}$. For each sample, patients that have the same activation pattern of features can be grouped together in the same subpopulation. For instance, subpopulation (1001) refers to all patients having the first and forth feature active. Let P refer to the total number of inferred subpopulations across the M posterior samples. By considering multiple posterior samples, we obtain slightly different partitions of patients in subpopulations. This can be seen as performing *soft-clustering* of patients, i.e., patients that are in-between subgroups might be assigned to different subpopulations in different posterior samples. Thus, the method is more robust against model inaccuracies at clustering patients. This is an important benefit of Bayesian modeling in general.

Next, in order to also make our method robust against outliers (patients with extreme biomarker values), we perform bootstrapping L times, for each subpopulation and posterior sample. Bootstrapping relies on random sampling with replacement. It is a technique used for computing robust estimators against outliers by sampling from an approximating distribution, which is particularly useful for hypothesis testing when the model assumptions are in doubt or unknown [10]. The standard bootstrapping approach relies on the construction of an estimator for hypothesis testing based on a number L of resamples with replacement of the observed dataset (and of equal size to the observed dataset), i.e., sampling with replacement from the empirical distribution of the observed data.

Given M posterior samples and L bootstrapping instances for each sample, we end up with ML different subpopulation instances. Measures of effect size (quantitative measure of the difference between two subpopulations) and statistical significance can be computed for each instance and then averaged across them, so that partition inaccuracies and outlier effects are mitigated. In the described algorithm, we suggest to compare each possible pair of subpopulations,[5] but we might want to focus only on the biggest communities or specific subpopulations of interest to reduce computational cost. Let Q be the total number of considered comparisons between subpopulations. In our particular case, $Q = P \cdot (P-1)/2$ as we consider each pairwise comparison among the P subpopulations. Let $i(q)$ and $j(q)$ refer to the set of subpopulation indexes corresponding to comparison q, e.g., $i(q) = 4$ and $j(q) = \{1, 2, 3\}$ corresponds to the comparison of subpopulation 4 against subpopulations 1, 2, and 3 aggre-

[4]Although Bayesian approaches to quantify statistical significance exist, such as posterior predictive checks or Bayesian factors, classical statistics predominate in the bio-medical field.

[5]Other comparison schemes are possible, such as a leave-one-out strategy consisting in the comparison of each individual subpopulation against the rest. Note that as the number of comparisons increase, the correction for multiple hypothesis testing shall be stronger.

gated. In the following, we will describe how to compute the $Q \times D$ effect size and statistical significance matrices.

Algorithm 2 Statistical approach for biomarker discovery (post-processing).

Input: M posterior samples from $\mathbf{Z}$ and $\mathbf{W}$, list of P subpopulations, and Q comparisons

```
for m = 1, ..., M do
    bootstrap for each subpopulation L times
end for
for q = 1, ..., Q do
    choose subpopulations G^{i(q)} and G^{j(q)}
    compute effect size according to Eq. 1, 2, and 3.
    compute statistical significance (p-value) according to the Mann-Whitney
    test for continuous variables and Fisher test for discrete variables, adjust-
    ing for multiple hypothesis testing [1]
end for
```

Output: effect size matrix $\mathbf{\Delta}$ and significance matrix $\mathbf{\Upsilon}$, both of dimensions $Q \times D$

Effect size. For each comparison q and dimension d, we compute the effect size Δ_{qd} as:

$$\Delta_{qd} = \mathbb{E}_{m,l}\left[\delta_{qd}(m,l)\right], \tag{1}$$

where $\boldsymbol{\delta}_{qd}$ is an $M \times L$ matrix of relative effect sizes for each posterior sample m and bootstrap iteration l. The expectation is done across all posterior samples and bootstrapping iterations, which are equally probable. In the case of continuous variables, we define

$$\delta_{qd}(m,l) = \log_2\left(\frac{\mu_d\left(\mathcal{G}_{ml}^{i(q)}\right)}{\mu_d\left(\mathcal{G}_{ml}^{j(q)}\right)}\right), \tag{2}$$

where $\mathcal{G}_{ml}^{i(q)}$ and $\mathcal{G}_{ml}^{j(q)}$ refer to subpopulations $i(q)$ and $j(q)$ in the posterior sample m and bootstrap iteration l, and $\mu_d(\mathcal{G})$ is the mean value of variable d within a given subpopulation $\mathcal{G}$. Taking the logarithm facilitates interpretation, such that an increase or decrease ratio has the same scale: for instance, $\delta_{qd}(m,l) = 0$ means that variable d has exactly the same averaged value in both subpopulations, $\delta_{qd}(m,l) = +1$ means that variable d is twice higher in subpopulation i, and $\delta_{qd}(m,l) = -2$ means that variable d is four times smaller in subpopulation $\mathcal{G}_{ml}^{i(q)}$ with respect to subpopulation $\mathcal{G}_{ml}^{j(q)}$. In the case of a discrete variable d, we check for mean differences, i.e.,

$$\delta_{qd}(m,l) = \mu_d(\mathcal{G}_{ml}^{i(q)}) - \mu_d(\mathcal{G}_{ml}^{j(q)}). \tag{3}$$

Note that we define different measures for continuous and discrete variables as the dynamic range of continuous variables is generally much higher, making the logarithmic scale more appropriate.

Statistical significance. To measure how significant an effect size $\delta_{qd}(m,l)$ is, for each posterior sample m and bootstrap instance l, we compute a statistical significance value $\upsilon_{qd}(m,l)$ as the p-value resulting from a certain two-sample test. In general, selecting the most appropriate statistical test in hypothesis testing is a challenging task [4, 5]. Here, we opt for one statistical test for all continuous variables and another one for discrete variables for simplicity, although more sophisticated strategies could certainly be investigated. We use the Mann-Whitney test for continuous variables and the Fisher test for discrete variables. The Mann-Whitney test is a general nonparametric statistical test to check whether the distribution of both populations are equal without requiring any normality assumption. The Fisher test is a standard test for categorical variables [10]. We define the $Q \times D$ matrix of statistical significance $\boldsymbol{\Upsilon}$, for each comparison q and biomarker d as the median p-value across the M samples and L bootstrapping instances:

$$\Upsilon_{qd} = \text{median}_{m,l}\left[\upsilon_{qd}(m,l)\right], \tag{4}$$

where $\boldsymbol{\upsilon}_{qd}$ denote the $M \times L$ matrix of statistical significance values $\upsilon_{qd}(m,l)$ for each posterior sample m and bootstrapping instance l. Finally, we follow the Benjamini Hochberg procedure for multiple hypothesis testing to adjust the statistical significance threshold α_s such that a certain false discovery rate is guaranteed [1]. A biomarker d is said to be statistically significant for comparison q if its significance value Υ_{qd} (the median p-value across posterior samples and bootstrapping instances) is smaller than the adjusted threshold, i.e., $\Upsilon_{qd} < \alpha_s$. Figure 3 illustrates the whole pipeline used in this paper.

References

[1] Yoav Benjamini and Yosef Hochberg. Controlling the False Discovery Rate: A Practical and Powerful Approach to Multiple Testing. *Journal of the Royal Statistical Society. Series B (Methodological)*, 57(1):289–300, January 1995.

[2] S. J. Gershman and D. M. Blei. A tutorial on Bayesian nonparametric models. *Journal of Mathematical Psychology*, 56(1):1–12, 2012.

[3] Thomas L. Griffiths and Zoubin Ghahramani. The Indian Buffet Process: An Introduction and Review. *Journal of Machine Learning Research*, 12:1185–1224, 2011.

[4] Vesna Ilakovac. Statistical hypothesis testing and some pitfalls. *Biochemia Medica*, 19(1):10–16, 2009.

[5] Marius Marusteri and Vladimir Bacarea. Comparing groups for statistical differences: How to choose the right statistical test? *Biochemia medica*, 20(1):15–32, 2010.

[6] Kevin P. Murphy. *Machine learning: a probabilistic perspective (Chap. 12).* MIT press, 2012.

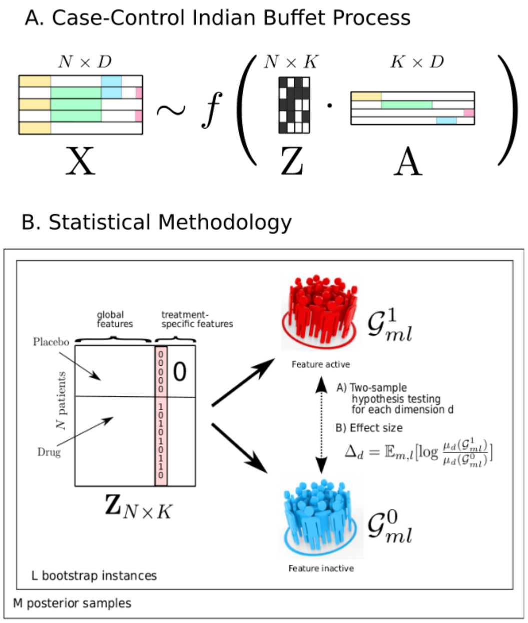

Figure 2: **Illustration of the pipeline used in this paper.** First, C-IBP finds a meaningful projection of the data, i.e., we identify patient subgroups according to the binary matrix **Z**. Second, classical statistical two-sample tests are performed by comparing patient subgroups for each learned latent feature.Illustration of the matrix factorization scheme.

[7] Isabel Valera, Melanie F. Pradier, and Zoubin Ghahramani. General latent feature modeling for data exploration tasks. *Workshop on Human Iinterpretability in Machine Learning at Neural Information Processing Systems*, 2017.

[8] Isabel Valera and Zoubin Ghahramani. General table completion using a Bayesian nonparametric model. In *Advances in Neural Information Processing Systems 27*, pages 981–989, 2014.

[9] Isabel Valera, Melanie F. Pradier, Maria Lomeli, and Zoubin Ghaharamani. General latent feature model for heterogeneous datasets. *Submitted to Journal of Machine Learning Research*, 2017.

[10] Larry Wasserman. *All of statistics: A concise course in statistical inference.* Springer Science & Business Media, 2013.
