# Supplementary material for: Case-control Indian buffet process identifies biomarkers of response to Codrituzumab
Source: BMC Cancer. 2019 Mar 28;19:278. doi: 10.1186/s12885-019-5472-0 (PMC6438135; doi:10.1186/s12885-019-5472-0)
Supplement: Supplementary file 4 — Relative effect size of biomarkers associated to each latent feature inferred by the C-IBP model. Significant biomarkers according to the Mann-Whitney test are marked with red circles. F1 identifies two types of patients with similar prognosis but different characteristics, F2 and F3 are associated with higher Progression Free Survival: F2 capture prognostic biomarkers while F3 capture predictive biomarkers. (PDF 85 kb) [file 12885_2019_5472_MOESM4_ESM.pdf]

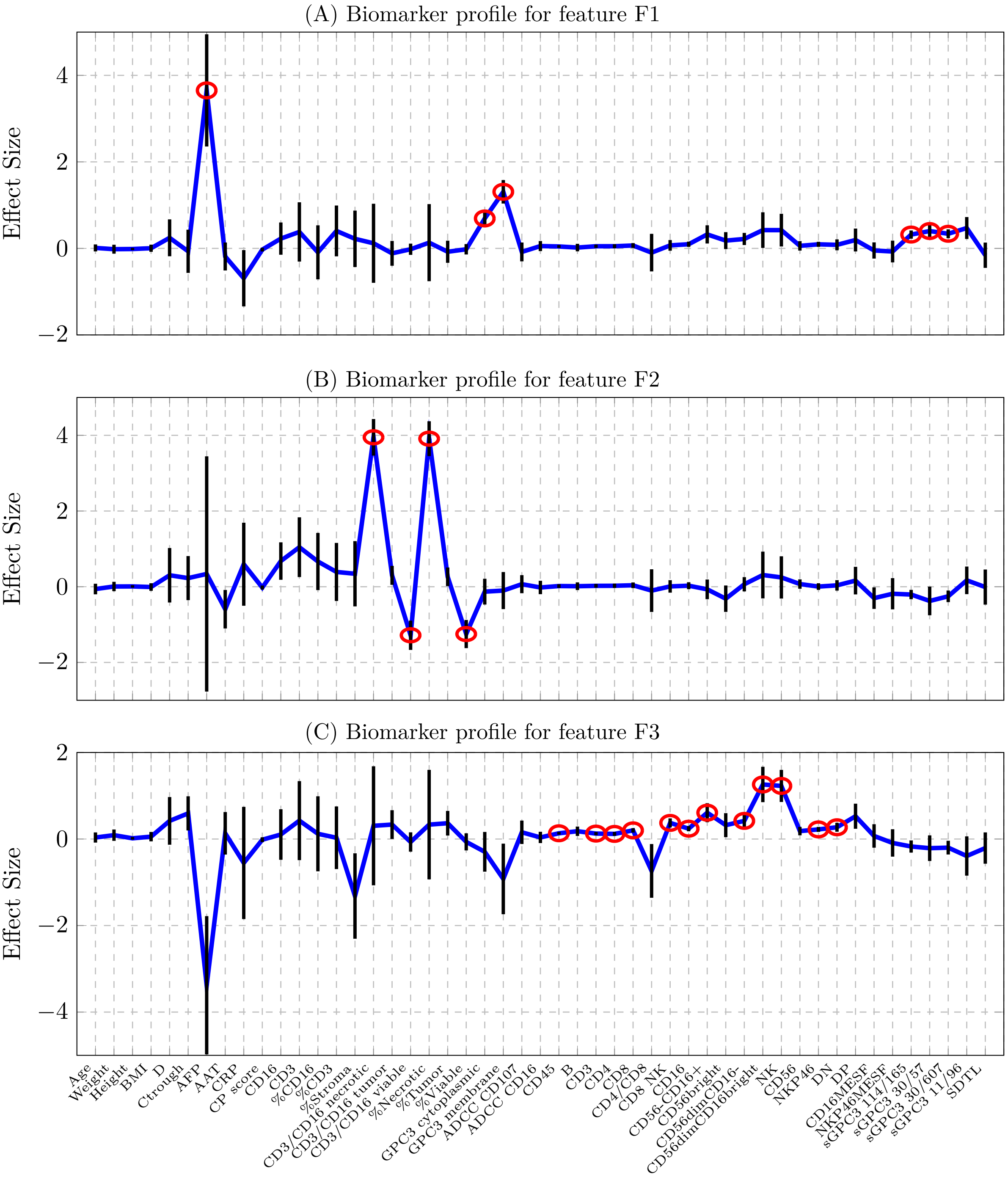
(A) Biomarker profile for feature F1
(B) Biomarker profile for feature F2
(C) Biomarker profile for feature F3
Effect Size
4
2
0
−2
−4
Age
Weight
Height
BMI
D
Ctrough
AFP
AAT
CRP
CP score
CD16
CD3
%CD16
%CD3
%Stroma
CD3/CD16 necrotic
CD3/CD16 tumor
CD3/CD16 viable
%Necrotic
%Tumor
%Viable
GPC3 cytoplasmic
GPC3 membrane
ADCC CD107
ADCC CD16
CD45
B
CD3
CD4
CD8
CD4/CD8
CD8 NK
CD16
CD56-CD16+
CD56bright
CD56dimCD16-
CD56dimCD16bright
NK
CD56
NKP46
DN
DP
CD16MESF
NKP46MESF
sGPC3 114/165
sGPC3 30/57
sGPC3 30/607
sGPC3 11/96
SDTL
